# Physical and Chemical Properties of *Acacia mangium* Lignin Isolated from Pulp Mill Byproduct for Potential Application in Wood Composites

**DOI:** 10.3390/polym14030491

**Published:** 2022-01-26

**Authors:** Nissa Nurfajrin Solihat, Eko Budi Santoso, Azizatul Karimah, Elvara Windra Madyaratri, Fahriya Puspita Sari, Faizatul Falah, Apri Heri Iswanto, Maya Ismayati, Muhammad Adly Rahandi Lubis, Widya Fatriasari, Petar Antov, Viktor Savov, Milada Gajtanska, Wasrin Syafii

**Affiliations:** 1 Research Center for Biomaterials, Research and Innovation Agency (BRIN), Jl. Raya Bogor KM 46, Cibinong 16911, Indonesia; karimahazizatul@gmail.com (A.K.); 103779316@student.swin.edu.au (F.P.S.); fayzaa_falah@yahoo.com (F.F.); maya_ismayati@brin.go.id (M.I.); marl@biomaterial.lipi.go.id (M.A.R.L.); 2 Department of Forest Products, Faculty of Forestry and Environment, IPB University, Bogor 16680, Indonesia; ekobudisantoso122@gmail.com (E.B.S.); elvarawindra@yahoo.com (E.W.M.); wasrinsy@indo.net.id (W.S.); 3 Department of Forest Product, Faculty of Forestry, Universitas Sumatera Utara, Medan 20155, Indonesia; apri@usu.ac.id; 4 JATI-Sumatran Forestry Analysis Study Center, Jl. Tridharma Ujung No. 1, Kampus USU, Medan 20155, Indonesia; 5 Faculty of Forest Industry, University of Forestry, 1797 Sofia, Bulgaria; p.antov@ltu.bg (P.A.); victor_savov@ltu.bg (V.S.); 6 Faculty of Wood Sciences and Technology, Technical University in Zvolen, 96001 Zvolen, Slovakia

**Keywords:** acid precipitation, single and fractionation step, kraft lignin, physical and chemical properties, *A. mangium* black liquor

## Abstract

The efficient isolation process and understanding of lignin properties are essential to determine key features and insights for more effective lignin valorization as a renewable feedstock for the production of bio-based chemicals including wood adhesives. This study successfully used dilute acid precipitation to recover lignin from black liquor (BL) through a single-step and ethanol-fractionated-step, with a lignin recovery of ~35% and ~16%, respectively. The physical characteristics of lignin, i.e., its morphological structure, were evaluated by scanning electron microscopy (SEM). The chemical properties of the isolated lignin were characterized using comprehensive analytical techniques such as chemical composition, solubility test, morphological structure, Fourier-transform infrared spectroscopy (FTIR), ^1^H and ^13^C Nuclear Magnetic Resonance (NMR), elucidation structure by pyrolysis-gas chromatography-mass spectroscopy (Py-GCMS), and gel permeation chromatography (GPC). The fingerprint analysis by FTIR detected the unique peaks corresponding to lignin, such as C=C and C-O in aromatic rings, but no significant differences in the fingerprint result between both lignin. The ^1^H and ^13^C NMR showed unique signals related to functional groups in lignin molecules such as methoxy, aromatic protons, aldehyde, and carboxylic acid. The lower insoluble acid content of lignin derived from fractionated-step (69.94%) than single-step (77.45%) correlated to lignin yield, total phenolic content, solubility, thermal stability, and molecular distribution. It contradicted the syringyl/guaiacyl (S/G) units’ ratio where ethanol fractionation slightly increased syringyl unit content, increasing the S/G ratio. Hence, the fractionation step affected more rupture and pores on the lignin morphological surface than the ethanol-fractionated step. The interrelationships between these chemical and physicochemical as well as different isolation methods were investigated. The results obtained could enhance the wider industrial application of lignin in manufacturing wood-based composites with improved properties and lower environmental impact.

## 1. Introduction

In 2019, Indonesia was ranked among the top 10 countries concerning pulp and paper production. In 2018, Indonesia produced 16 million tons of paper and 11 million tons of pulp [1]. For every 1 ton of pulp produced, about 7 tons of black liquor (BL) were generated as a residue at 15% solids by weight, with two-thirds of the solids consisting of organic chemicals, and the remains were inorganic chemicals [2]. In recent decades, lignin derived from BL has been considered a natural biopolymer, a viable alternative to the fossil-based chemicals due to its abundance in BL, reaching 45% dry weight [3].

Most BL is incinerated for boiler heating sources and energy, and only 5% of the BL is used for value-added applications [4]. The economical consideration of lignin isolation from BL includes recovery yield, purification, non-uniform structure, and unique reactivity [5]. The three main phenolic hydroxyl precursors in lignin are coniferyl alcohol (G), p-coumaryl alcohol (H), and sinapyl alcohol (S), which are linked to each other mostly by aryl ether linkage (β-O-4′) [6,7]. The actual properties of lignin, such as thermal stability, reactivity, molecular distribution, and solubility, depend on the ratio of these aromatic units. It varies depending on the technique of extraction and the plant source. For instance, softwoods contain mostly G units; hardwoods include both S and G, while non-wood plants have all three units [8].

Precipitation by dilute acid such as sulphuric acid is a common and feasible technique to isolate lignin from BL [9]. However, using sulphuric acid can increase lignin’s ash and sulfur content. Therefore, lignin for sulfur-sensitive utilizations should be restricted [5]. Haz et al. evaluated the effect of four different dilute acids (chloric, sulphuric, acetic, and nitric) on lignin properties. Lignin precipitated by nitric and chloric acid obtained high phenolic hydroxyl both in non-conjugated and conjugated forms (>2 mmol/g), suitable for phenolic polycondensates production and rubber stabilizer [10]. Handika et al. [11] reported that high free-phenolic hydroxyl in lignin increased its reactivity to produce the high-thermal stability of polyurethane resin for textile application. As a polyphenol molecule, lignin contains high free-phenolic hydroxyl groups that are favorable for modifications, such as phenolation and methylolation [12], tailored for increasing its chemical reactivity to formaldehyde in formaldehyde-based resins used in the production of wood composites such as particleboards [13], oriented strand boards [14], and flame-retardant composites [15]. Besides, lignin modification either with poly(butylene succinate) or polypropylene biocomposites increased the thermal stability of kenaf core fiber [16,17]. According to Tejado et al., kraft lignin is suitable for phenol-formaldehyde resin due to its higher amount of free phenolic content, molecular weight, and thermal properties [18]. Therefore, it is necessary to understand the specific chemical structure of lignin to achieve optimal utilization. However, the lack of understanding of the lignin structure-property–application relationship (SPARs) is a major roadblock to further development of lignin [19]. Of these, one characterization technique is insufficient to produce coherent data to identify the feature of lignin because of its complex structure and variation. Therefore, a comprehensive analytical technique is pivotal to understanding the properties of isolated lignin, allowing its large-scale utilization.

This study emphasizes the efficient isolation method of lignin from industrial residues of the pulp and paper industry. Our fundamental comprehensive analytical technique provides knowledge for industries to produce superior lignin-based, value-added products, especially for wood composites. Lignin was isolated by two different methods of dilute chloric acid precipitation. The physical characteristics of lignin, such as morphological structure, were conducted by scanning electron microscopy (SEM). Chemical features, including its total hydroxyl phenolic content and solubility in the organic and base solvent, were determined by ultraviolet-visible spectrophotometer (UV-Vis). The functional group was identified by attenuated total reflection Fourier-transform infrared (ATR-FTIR). ^1^H and ^13^C nuclear magnetic resonance (NMR) were employed to predict structural properties of lignin corresponding to its fingerprint signal. The elucidation aromatic precursors unit in lignin structure was analyzed by pyrolysis-gas chromatography-mass spectrometry (PyGC/MS). Meanwhile, the thermal features of lignin were characterized by thermogravimetric analysis (TGA) and differential scanning calorimetry (DSC). Gel permeation chromatography (GPC) was used to measure the molecular distribution of lignin.

## 2. Materials and Methods

### 2.1. Material

A derived BL from *Acacia mangium* was collected from a pulp and paper mill factory in Sumatra, Indonesia. Hydrochloric acid (HCl), dioxane, sodium hydroxide (NaOH), acetic acid anhydride, dimethyl sulfoxide (DMSO), and tetrahydrofuran (THF) were purchased from Merck (Darmstadt, Germany), while pyridine was obtained from Wako Pure Chemical Industries (Osaka, Japan). Lignin alkali (kraft) from Sigma-Aldrich (Saint Louis, MO, USA) was used for the lignin standard. All chemical materials used in this study were analytical grade without any purification.

### 2.2. Lignin Isolation

Dilute acid precipitation was used to isolate lignin from BL through a single-step and fractionated-step based on Hermiati et al. [20] with one major modification. For the single step, HCl 1 M was poured into BL until pH 2. The solution was kept overnight and the residue was separated by decantation. The deionized (DI) water was added to the residue with similar acid volume, and the decantation process was conducted again after 24 h. This process was repeated six times. The residue was kept in the refrigerator overnight and separated by vacuum filtration. Wet lignin on the filter paper was washed with DI water and dried in an oven at 45 °C for 24 h. The lignin yield percentage was measured by dividing the dry weight of lignin (g) by the dry weight of BL. Dried lignin was kept in sealed plastic for further analysis.

For fractionated-step, HCl 1 M was added into BL until pH 7. Ethanol, as much as four times the volume of the acid, was added to the solution. Non-lignin components such as sugar and carbohydrates were filtrated as a residue. The filtrate was evaporated until the ethanol completely dried up. The acidification was continued by adding acid until pH 2. Lignin precipitate was separated without decantation six times by water, unlike the single-step. The following step is similar to the procedure from the single-step method.

### 2.3. Chemical Features Measurement: Chemical Component, Total Phenolic Hydroxyl, and Solubility

The water content of lignin was determined according to TAPPI T 264 cm-97 [21], and ash content was calculated following the TAPPI T211 om-02 method [22]. Contents of acid-insoluble lignin (AIL) and acid-soluble lignin (ASL) were analyzed based on the method of Sluiter et al. (NREL/TP-510-42618) [23]. Triplicate analysis was performed for all chemical features measurements. Lignin alkali (kraft) from Sigma-Aldrich (370958) (Saint Louis, MO, USA) was used as a reference.

Total phenolic hydroxyl (phOH) was determined by the UV-Vis method [24]. A total of 1 mg/mL of lignin was diluted in dioxane: 0.2 M NaOH (1:1), and the mixture was filtered by microfiltration (0.45 µm). The filtrate was diluted in 0.2 M NaOH until the 0.08 mg/mL concentration was reached. The UV spectrum was recorded in a 200–600 nm range by Shimadzu UV vis-1800 spectrophotometer, where lignin in pH6 was used as reference. The absorbance of maximum spectra at 300 and 350 nm was used to calculate total phOH by the following equation:

$$Total\;phOH\;\left(\mathrm{mmol}/g\right)=\left(0.425\times A_{300\;\mathrm{nm}\;}\right)+\left(0.812\times A_{350\;\mathrm{nm}\;}\right)\times \frac{1}{c\times a}\times \frac{10}{17}$$

where *A* is absorbance, *c* stands for lignin concentration, and *a* is path length (1 cm) [24].

The solubility test of lignin in the base and the organic solvent was conducted according to the method by Hermiati et al. Lignin 7 mg/5 mL was dissolved in NaOH pH 12 as the alkali solvent and mixture of dioxane water (9:1). Each solution was diluted 50 times by DI water. The UV spectrum was measured in the range of wavelength 200–400 nm [20].

### 2.4. Morphological Assessment by SEM

A scanning electron microscope (JSM-IT200, JEOL, Tokyo, Japan) was used to observe morphological surfaces and particle size of the reference lignin and the isolated lignins. Lignin samples were placed on the carbon tube, and the surface was coated with gold using Ion Coater iB2. The micrograph of the sample was recorded at 200 and 5000 magnifications under a high vacuum and working distance of 11 mm with 5.0 kV accelerating voltage.

### 2.5. Functional Group Analysis by UATR-FTIR

Attenuated total reflection Fourier-transform infrared (ATR-FTIR) spectroscopic equipped with UATR unit cell from PerkinElmer (spectrum two) (PerkinElmer Corporation, Waltham, MA, USA) was employed to investigate the functional group of lignin. The sample was placed on the diamond crystal, and the spectrum at a wavelength of 400–500 cm^−1^ was taken by pressing the torque knob with the same pressure. An average of 32 scans with 4 cm^−1^ resolution were used to acquire the spectrum. The same average scanning was carried out for background correction and scanning before analysis.

### 2.6. Fingerprint Observation by 1H and 1C NMR

Lignin samples were acetylated before undergoing nuclear magnetic resonance (NMR) and molecular weight distribution test based on Wen et al. [25] method with a minor adjustment. A total of 200 mg lignin was dissolved in an 8 mL mixture of acetic acid anhydride: pyridine (1:1) for 72 h in the dark bottle. Ethanol was added until the mixture was concentrated. Acetylated lignin was obtained by slowly dropping the mixture into ice acid (pH 2) and separated through centrifugation. The wet acetylated lignin was washed with 50 mL DI water three times and freeze-dried until dry acetylated-lignin (AL) was obtained.

Each 20 mg AL sample was diluted in 0.7 mL DMSO. The solution was transferred to a 3 mm tube. ^1^H NMR (JEOL JNM-ECZR 500, Tokyo, Japan) data points were acquired with an acquisition time of 1.75 s, a relaxation time of 5.0 s, and 24 scans. For typical ^13^C NMR, 20,480 spectra scanning were averaged to increase the signal-to-noise ratio with 2.0 s delayed relaxation and 0.9 s acquisition time.

### 2.7. Thermal Investigation by TGA and DSC

The thermal investigation of the isolated lignin was conducted using a thermogravimetric analyzer (TGA 4000, PerkinElmer, Waltham, MA, USA) and differential scanning calorimetry (DSC) (DSC 4000, PerkinElmer, Waltham, MA, USA). For TGA analysis, about 4 mg lignin sample was placed on the crucible ceramics sample holder, and the measurement was conducted under argon atmosphere with the flow of 20 mL/min. The sample was heated from 25 °C to 750 °C at a 10 °C/min rate. The automatic curve of weight loss versus temperature was generated from the instrument.

DSC analysis was carried out with ~4 mg lignin samples on a standard aluminium pan to determine the glass transition temperature (Tg) and curing properties of lignin. Each sample was heated until 300 °C with a 10 °C/min heating rate under a nitrogen atmosphere (flowrate = 20 mL/min). Tg value was automatically calculated by DSC 4000 pyris 1 PerkinElmer software (Pyris 11 software Version 11.1.1.0492, PerkinElmer, Shelton, CT, USA).

### 2.8. Chemical Elucidation by Py-GCMS

Chemical elucidation analysis of lignin was studied by pyrolysis-gas chromatography-mass spectrometry (PyGC/MS) (Shimadzu GC/MS system QP-2020 NX, Shimadzu, Kyoto, Japan) equipped with multi-shot pyrolyzer EGA/PY-3030D. Between 500–600 µg of lignin was placed in eco-cup SF PY1-EC50F, and the cup was sealed by glass wool. The eco-cup was pyrolyzed at 500 °C for 0.1 min using helium as carrier gas and SH-Rxi-5Sil MS column (30 m × 0.25 mm i.d. film thickness. 0.25 µm). The PyGC/MS temperature was programmed as follows: 50 °C for 1 min, 5 °C/min to 280 °C, and 13 min at 280 °C. The mass spectrum was taken at 70 eV with a pressure of 20.0 kPa (15.9 mL/min, column flow 0.61 mL/min). The obtained pyrolysis product was identified by approaching mass spectra and retention times using the data library in NIST LIBRARY 2017.

### 2.9. Molecular Weight by GPC

Gel permeation chromatography (GPC) is a rapid and versatile tool to provide information on the molecular weight of lignin. Lignin was dissolved in the THF, and Shimadzu LC-20 (Shimadzu, Kyoto, Japan) equipped with a UV-RID detector was used to quantify the molecular weight distribution acetylated lignin. The analysis was employed using the LF-800 column with an injection volume of 20 uL. Polystyrene standard was used to create a calibration curve and GPC system calibration.

## 3. Result and Discussion

### 3.1. Chemical Composition and Lignin Solubility

Lignin recovery was one critical factor for selecting the lignin isolation method that is economically feasible. Lignin yield recovery from BL by single and fractionated-step (oven dry based) was 35.39% and 16.34%. Both isolation methods reported lignin recovery yield at the expected range of 20–40% [5]. Ethanol fractionation resulted in lignin depolymerization in the liquid solution, decreasing the solid lignin residue by acid. Lignin yield recovery is related to the larger size of the fractionated-step lignin based on the SEM micrograph. Large lignin particle size results in a smaller reaction surface area for precipitation, reducing the amount of lignin recovered after acid precipitation. This suggestion was in correlation with the ASL content. This finding agreed with Hamzah et al. (2020), where lignin recovery from *Miscanthus x giganteus* decreased from 75% to 25% with the increased ethanol concentration from 0% to 50% [26].

The chemical composition of lignin Is presented in Table 1, where the data is the average value from a triplicate experiment with a deviation standard less than ±5%. Ethanol was added in fractionated-step to precipitate non-lignin components such as sugar and carbohydrates, theoretically increasing lignin purity. However, the total lignin from single-step lignin (~99%) was slightly higher than from the fractionated step. Besides, the impurities component in the single-step lignin, represented as ash content, was lower than the fractionated step. It suggests that the single-step isolation method effectively isolates lignin with high purity.

Interestingly, lignin isolation from BL by dilute hydrochloric acid obtained high ASL content while reference lignin had low ASL content. Different isolation methods likely obtained the different proportions of AIL and ASL content. Similar results were reported by Sameni et al. [8] where isolation lignin from BL by using dilute sulfuric acid resulted in low ASL (<4%) and high AIL ~91. Sulfuric acid is popular acid to isolate lignin and obtain a high concentration of AIL. Consequently, it will increase sulfuric and ash content [5].

In this study, the highest free phOH content was obtained from the isolated lignin, where the lowest was from the reference lignin. This finding agreed with the previous report where Kraft pulping process and precipitation lignin by HCl enhanced condensed structure and the phenolic hydroxyl group [10,27]. Unfortunately, we could not find the source and isolation process of reference lignin from Sigma-Aldrich. The total phOH content is correlated to the Tg value because a higher condensed structure in polymer created a high char amount in high temperature. Eventually, the combustion rate can be reduced by the presence of char [11,28,29].

**Figure 1:**
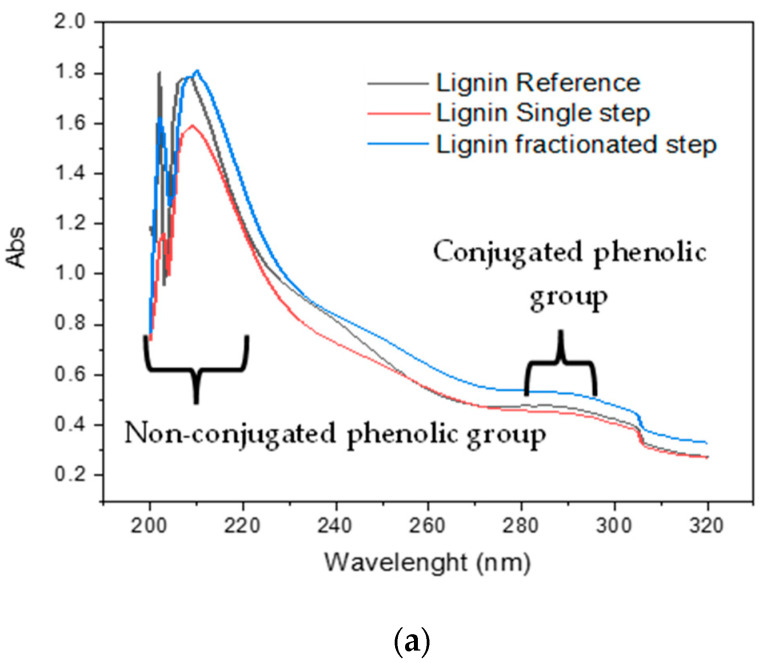

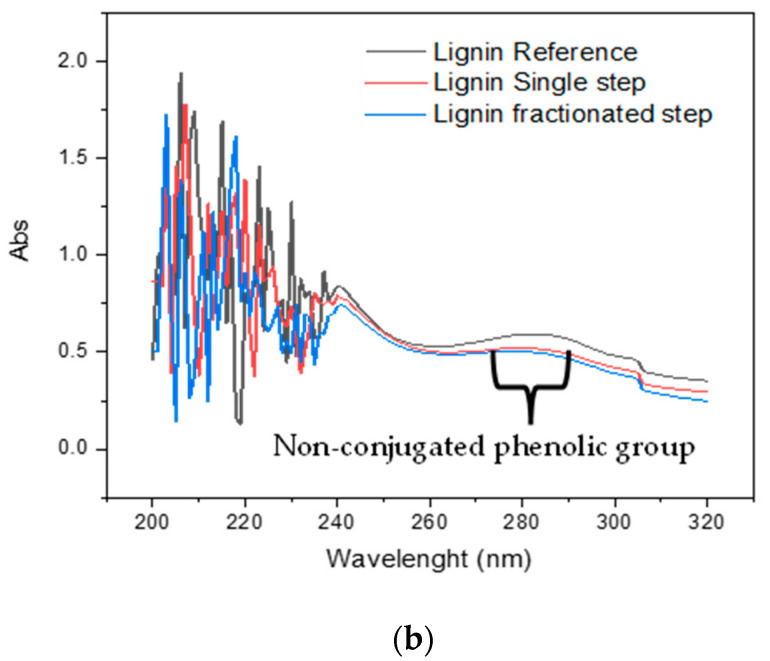
Lignin solubility in base (**a**) and organic solvent (**b**) determined by UV-Vis.

UV spectroscopy is used to monitor the lignin purity and molecular distribution. A similar pattern of UV-Vis spectra from both commercial and isolated lignin is observed in Figure 1a. The distinct absorption at 215–222 nm corresponded to non-conjugated phenolic groups (excitation of π-π*) that appear due to shifting band is an effect of hypsochromic NaOH. The spectrum of single-step lignin is slightly higher than others which may correlate to the total phOH (Table 1). Another intensive peak was observed in the range of 296–303 nm, originating from the conjugated phenolic group due to n-π* excitation [30].

A glance at Figure 1b reveals an identical UV-Vis spectrum among three lignin samples when lignin is diluted in dioxane/water. Unlike the lignin in the base solution, solubilization lignin in dioxane/water is limited to wavelengths above 250 nm seen in the spectra Figure 1b. This finding is similar to lignin Alfa grass kraft from industrial waste [31] and Kraft-anthraquinone (AQ) lignin [32]. According to Ammar et al.’s report, the large absorbance of lignin in dioxane/water at 280 nm corresponded to non-conjugated phenolic hydroxyl groups. In comparison, the presence of both ferulic acids and p-coumaric acids could be attributed to the presence of the second type region of lignin absorption at about 300 nm [31]. Lignin reference has slightly higher absorbance than isolated lignin regarding purity.

### 3.2. SEM Micrograph of Lignin

**Figure 2:**
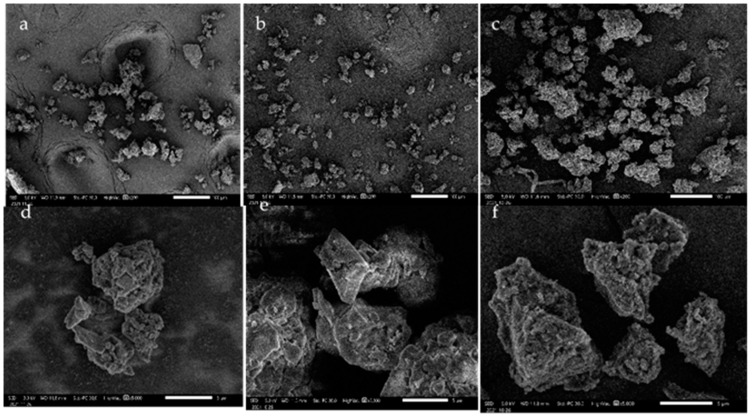
SEM micrographs show the morphological surface of reference lignin (**a**), single-step (**b**), fractionated-step (**c**) at 200× magnification and reference lignin (**d**), single-step (**e**), and fractionated-step (**f**), at 5000× magnification.

SEM micrograph in Figure 2a–c, shown in 200-times magnification, described irregular and not uniform particles in terms of size from lignin samples. The reference lignin had a smaller particle of 59 µm than lignin from fractionated-step isolation (~72 µm). Interestingly, the single-step lignin had the lowest particle size, ~51 µm, indicating that ethanol impacted the behavior of lignin aggregates. At 5000-times magnification (Figure 2d–f), the morphological image of reference lignin (Figure 2d) and lignin from a single-step (Figure 2e) showed more rupture and pores on the lignin surface. Lignin from fractionated-step (Figure 2f) depicted smooth and rigid surfaces. This finding was following lignin isolation from *Miscanthus x giganteus* where the particle size of lignin increased with higher ethanol concentration, from 306 to 2050 nm. Besides, more crystalline structures and pores on the lignin surface were observed in more concentrated ethanol [26].

### 3.3. Functional Group of Lignin

**Figure 3:**
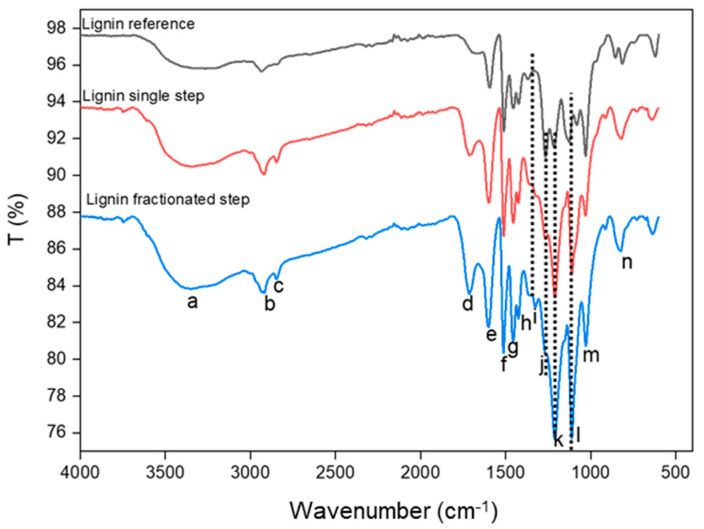
Functional group peaks of reference lignin, isolated lignin from single-step and fractionated-step by UATR-FTIR.

FTIR is a versatile analytical tool to investigate functional groups and the general structure in lignin. The functional group of lignin may vary depending on the source of lignin. The main functional group in lignin is hydroxyl, methoxyl, carboxylic acid, and carbonyl. Figure 3 shows the FTIR spectra of reference lignin, and two isolated lignin, while a summary of peaks interpretation is available in Table 2. As shown in Figure 3, most of the peaks were similar between three samples, such as the broadband corresponding to hydroxyl group stretching (O-H) from aliphatic and aromatic in lignin structure detected at the wavelength 3500–3400 cm^−1^ (a), while the sharp peak at 2918 (b) and 2854 (c) cm^−1^, respectively, were attributed to C-H stretching in methylene from side chain and aromatic methoxyl groups [33]. A small band assigned to carbonyl (C=O) stretching in unconjugated aldehyde and ketone in the ester group at 1716–1704 cm^−1^ (d) was found in the reference lignin. Still, a pronounced peak was obtained from both isolated lignin due to different lignin structures. Noticeable peaks attributed to vibration of the aromatic skeleton in all types of lignin appeared in the range of 1590–1460 cm^−1^ (e–g) [34]. An intense peak at 1430–1420 cm^−1^ (h) referred to aromatic skeletal vibration with deformation of C-H asymmetric in a methyl group [4].

However, an obvious difference in lignin structure between reference and isolated lignin was observed in adsorption at 1326 cm^−1^ (i) and 1111 cm^−1^ (l) as the breathing of C-O and deformation C-H in syringyl rings. The band was absent in the reference lignin, but it appeared in two isolated lignins. Conversely, stronger C-O stretching in the guaicyl unit at 1266 cm^−1^ (j) and 1213 cm^−1^ (k) was recorded in reference lignin but not in isolated lignin since the lignin was extracted from *Acacia mangium* (hardwood). This finding suggests that the reference lignin may be derived from softwood. This result correlates with Sameni et al. [35] finding where syringyl unit portion was absent in lignin from softwood and abundant in lignin from hardwood. Furthermore, higher peak absorption of unconjugated C-O (at 1030 cm^−1^ (m)) and CH out-of-plane bending (at 855 cm^−1^ (n)) in the guaicyl ring in reference lignin suggests a higher concentration of guiacyl in softwood than hardwood. Besides, these peaks were also slightly sharper in the lignin fractionated-step than in the single-step. Further semi-quantitative analysis of syringyl versus guaicyl percentage is described in the Py-GCMS section.

### 3.4. ^1^H and ^13^C NMR

NMR analysis is frequently used to predict lignin’s structural details concerning its molecular characteristics, reactivity, and composition. The acetylation of lignin before NMR analysis aims to decrease the impurities in lignin that may interfere with the spectrum [25]. Due to the complex structure of polymer lignin, typically simple proton ^1^H NMR resulted in overlapping spectra which is difficult to justify the structure. Hence, ^13^C NMR is needed to support the hypothesis of ^1^H NMR. The presence of condensed and uncondensed aliphatic and aromatic carbon and aryl ethers can be detected by natural ^13^C isotope NMR. However, longer scanning and acquisition times are required to improve signal sensitivity due to the low abundance of carbon isotope in the lignin molecule [4]. Still, quantitative ^13^C-NMR can be a useful technique for lignin structural investigation, particularly in determining molecular alterations caused by different isolation procedures and biomass sources [8,25,35].

**Figure 4:**
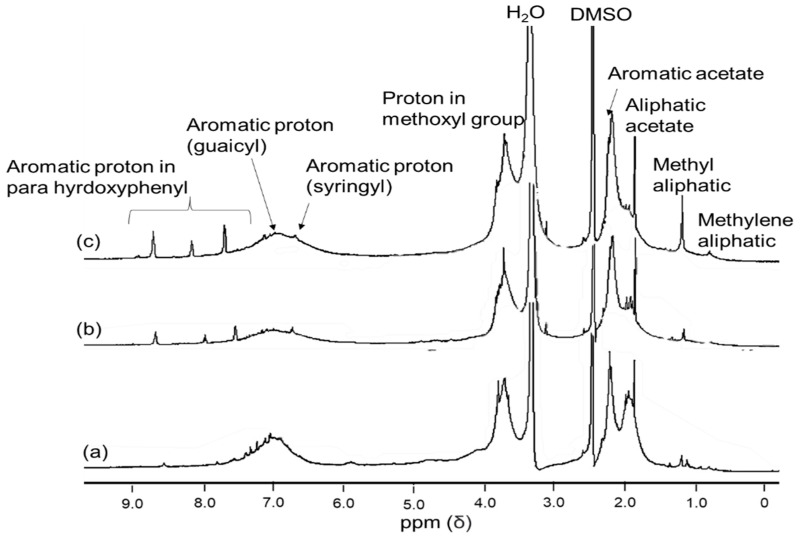
^1^H NMR signal of standard lignin (**a**), single-step lignin (**b**), and fractionated-step lignin (**c**).

The ^1^H NMR spectrum in Figure 4 shows similar peaks among the three lignin samples. The small peak at 0.8 and 1.23 ppm occurred because of saturated aliphatic lignin protons in the methyl and methylene chain. The intense signal at 1.98 ppm indicates the presence of an aliphatic acetate group. The strong signal at 2.5 ppm and 3.3 is because of protons in water and DMSO. A pronounced peak at 3.76 ppm corresponds to methoxyl protons (-OCH_3_). A sharper signal at 6.7–6.9 in isolated lignin spectrum suggests more syringyl units than reference lignin.

Conversely, a more intensive peak at 7 ppm was found in reference lignin due to the higher guaicyl content of reference lignin than isolated lignin. This trend agrees with the FTIR and Pyr-GC/MS results, where isolated lignin has more syringyl units than reference lignin. The other strong signal in the range of 7.5–8.5 ppm reveals an aromatic presence in p-hydroxyphenyl proton positions 2 and 6 [38]. The obtained signal in the NMR spectra of isolated lignin was similar to lignin from sweet sorghum stem (SST) [34] and lignin kraft [8]. Nevertheless, the peak spectrum was slightly different with lignin from Ginkgo shells, where the Hα signals at 5.5–5.9 ppm related to linkages of β-O-4′ and β-5′ were produced [39]. The results may reveal that different sources of lignin generated different structures.

**Figure 5:**
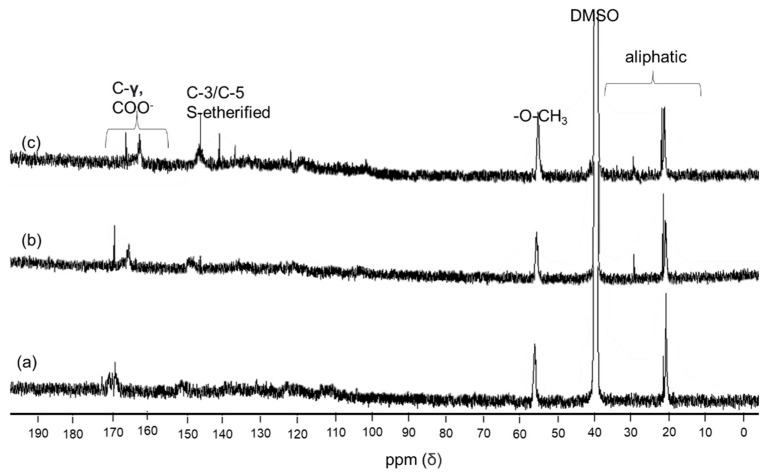
^13^C NMR spectra of standard lignin (**a**), single-step lignin (**b**), and fractionated-step lignin (**c**).

The ^13^C NMR spectra (Figure 5) shows a unique signal related to the lignins in this study. All the lignin samples show a similar trend of peaks where five typical lignin signals show strong resonance such as aliphatic chains, solvent (DMSO), methoxy, C3/C5, and ester. The signal in region between 20–30 ppm represents an aliphatic chain structure in lignin where isolated lignin (b–c) has a stronger signal than commercial lignin (a), which corresponds with FTIR spectra (2918 and 2854 cm^−1^) and ^1^H NMR (0.8 and 1.23 ppm). Meanwhile, the strong peak at 40 ppm belongs to DMSO as a solvent. The intensive signal at 55.9 ppm is attributed to the methoxy group in the G and S units. A signal related to esterified syringyl unit in C3/C5 is observed at 152 ppm in isolated lignin (b–c) but is not detected in reference lignin (a). Repeatedly, this result agrees with FTIR, Py-GC/MS, and ^1^H NMR. A strong signal at a 170–160 ppm range implies ester linkage (-COO^−^) at γ position [34].

In general, the obtained signal in this study was also detected in lignin from sweet sorghum stem SST [34] and lignin kraft from the industrial residue [32]. However, the assigned peak correlated to the G unit is not detected in this current spectrum. it is likely that this is caused by either the lignin concentration being too dilute or the presence of the Nuclear Overhauser Effect (NOE). According to a report by Wen et al. [40], an important aspect of lignin characterization fulfills three criteria. First, lignin should be free from impurities; for this case, acetylation improved the purity of lignin, which was proved by residual carbohydrate’s absence signal at 62, 73–75, and 100–102 ppm [25]. Second, lignin solution must be concentrated to minimize baselined phasing distortion and increase the signal-to-noise ratio, yet this requirement negatively affects the LC column. Third, to avoid the NOE, the inverse-gated decoupling sequence (i.e., C13IG pulse) should be utilized, which entails turning off the proton decoupling during the recovery between pulses [40].

### 3.5. Thermal Behavior of Lignin

**Figure 6:**
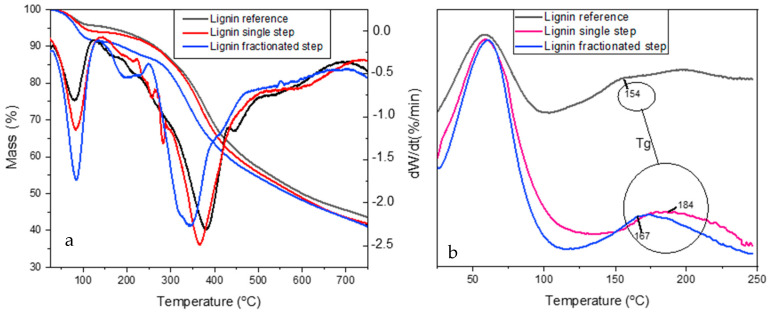
TGA (**a**) and DSC (**b**) thermogram of lignin standard, single-step lignin, and fractionated-step lignin.

Mass loss (TG) and mass loss rate (DTG) curves of *A. mangium* lignin are shown in Figure 6a to indicate the similar thermal characteristics between isolated lignin and reference lignin. The reaction region of all stages shifts toward a higher temperature by increasing the heating rate for isolated and reference lignin. The primary loss stage of two isolated lignins and their reference was located in a broad temperature range (between 100 °C and 700 °C), representing a complex structure consisting of phenolic hydroxyl, carbonyl benzylic hydroxyl functionalities [41]. The decomposition of lignin by temperature can be divided into three stages. The initial pyrolysis stage at around 100 °C with a higher mass loss was represented by the fractionated-step lignin. This first stage, up to 200 °C, is mainly attributed to the moisture evaporation in lignin and releasing of volatile products such as carbon dioxide and carbon monoxide [30]. A similar degradation study of lignin reported that an endothermic peak ranges from 100–180 °C, corresponding to the elimination of humidity [42].

The second pyrolysis stage, between 120 °C and 270 °C, indicated the decomposition of lignin into some possible degradation products and the removal of carbohydrates from lignin. The peak of the stage was around 200 °C, and below this peak, lignin is thermally stable. The losses in this stage were derived by aromatic decomposition as a phenolic compounds, such as the cleavage of ether linkages among the C9 units [43]. Thermal degradation of lignin is followed by condensation processes, leading to unsaturated C=C bonds occurring in the temperature range of 160 to 270 °C. Afterward, the production of vinyl guaiacol, ethyl, and methyl byproducts is usually obtained at 230 and 260 °C with the degradation of the propanoic side chains of lignin [41].

The third pyrolysis stage had a temperature range of 270 °C to 700 °C, with the prominent peak being around 350 °C and 380 °C for the fractionated-step lignin and single-step, respectively. The reference lignin reached the highest peak, indicating it as the most stable. The lignin structure is decomposed majorly at a temperature of 260–478 °C. At temperatures below 310 °C, aryl ether links tend to cleave, caused by low thermal stability [44]. At higher temperatures (>500 °C), aromatic structures rearranged and condensed the lead into char [44,45] and released volatile products. The high capacity to produce char by lignin makes it an efficient alternative to improve the flame retardancy of polymers [7]. Similar tendencies were also observed in other studies concerning the mass loss and the evolution of the volatiles against the origin and pyrolysis temperature [46]. Hu et al. [47] studied the isolated lignins extracted by different solvents and reported that CH_4_, CO, and phenols are lignin’s main mass loss stage. Based on TGA analysis in the third stage, the fractionated-step lignin was decomposed at a lower temperature with moderate mass loss than the single-step lignin due to the extraction method used. This result indicates that fractionated-step lignin yields better purity than single-step lignin. The mass loss in this third stage was remarkable over in the second stage for isolated lignin and reference lignin, attributed to volatiles’ intensive evolution in the third stage [43].

The glass transition temperature (T_g_) of the lignin fractionated-step and single-step was higher than that of reference lignin, with T_g_ temperatures of 184, 167, and 154 °C, respectively (Figure 6b). Furthermore, the first peak at 50 °C indicated an endothermic process in which the lignin absorbed heat energy to evaporate water and other volatile substances [48]. T_g_ represents the end of an endothermic process in which the lignin structure changes from a glassy state into a rubbery (plasticized) state. The wide range of T_g_ values indicated the flexibility and stiffness at higher temperatures, beneficial in industrial applications [35]. The T_g_ value varies widely depending on the method of lignin isolation, adsorbed water, molecular weight, and thermal history [49]. Since single-step lignins with higher T_g_ values are more stable at high temperatures, the process requires higher temperature operation.

The higher Tg value of isolated lignin compared to reference lignin was due to the higher amount of phOH content in the isolated lignin (Table 1). Intramolecular hydrogen bonds between phOH groups in the main back bonds of lignin contributed to the higher T_g_. The bonds created a physically cross-linked structure [50]. The T_g_ value is influenced by the solubility of the organic solvents, where higher solubility is obtained with a lower T_g_ value [51,52]. The reference lignin had a higher absorbance in dioxane, the organic solvent, than isolated lignin, as presented in Figure 1b (solubility lignin in dioxane). This finding is supported by the lower T_g_ value of reference lignin than the isolated lignin and agrees with the results reported by Dastpak et al. [52] where Kraft lignin had higher T_g_ and lower solubility in the organic solvent than organosolv lignin. The T_g_ value corresponds positively with the molecular mass of lignin [53,54,55]. The T_g_ shifts to higher temperatures by increasing the average molar mass [56]. Based on the T_g_ value, the fractionated-step lignin had a lower molecular mass than the single-step lignin. This might be caused by the more extended process obtained by using acid precipitation to condense lignin, continued by ethanol addition to adsorbing the carbohydrate attached to lignin during the kraft pulping process. This suggestion was supported by GPC analysis in the next section.

Impurities influenced the T_g_ value in the lignin sample represented by ash content. The fractionated-step lignin and reference lignin had higher ash content (1.96% and 2.58%, respectively) compared to single-step lignin (0.48%), resulting in a lower T_g_ value. Sameni et al. (2013) also reported a higher percentage of impurities obtained with a lower T_g_ value. The abundance of aromatic rings in the main backbone of lignin can also contribute to the higher T_g_ value of isolated lignin. The varied T_g_ values were due to the heterogeneous structures and the broad molecular weight of isolated lignin samples [57]. These factors also affect interchain hydrogen bonding, cross-linking density, and rigid phenyl groups [58]. Although several studies reported an increase of char residue with higher Tg values, the results may be inconsistent due to the plant sources and extraction conditions [35]. The two isolated lignins from *A. mangium* in this study had higher T_g_ values in comparison to the others hardwood lignin, for instance T_g_ value from *Eucalyptus Grandis* was 161 °C [59], while eucalyptus kraft lignin was 133 °C [60], and other hardwood kraft lignin showed values such as 108 °C [61] and 138 °C [62]. pH solution conditions also influenced the T_g_ during lignin precipitation that varied from 106.12–131.81 °C at pH 1–5 [55].

### 3.6. Chemical Elucidation by Mass Spectrometry

**Figure 7:**
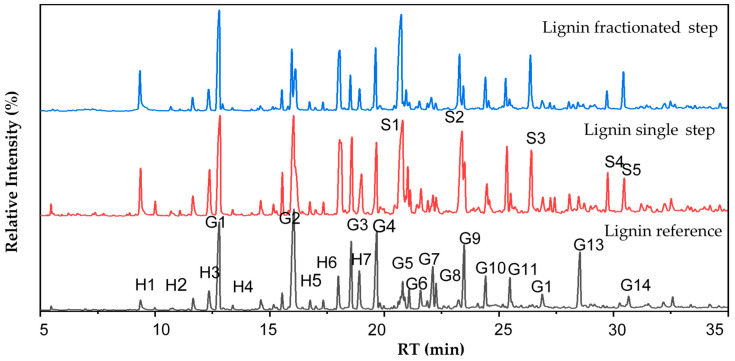
Pyrogram of single-step lignin and fractionated-step lignin compared to reference lignin.

Py-GCMS helps determine the lignin degradation and the existence of carbohydrates and other additives [63]. The PyGCMS method can help unravel the nature of lignins, elemental composition, number of formed products, and the isolation method [64]. The monomer unit in hardwood lignin consists of syringyl (S) and guaiacyl (G) units with varying proportions, while softwood is dominated by a high proportion of G units with less p-hydroxyl phenyl (H) units [65]. In native wood, higher S units are easier to delignify than lower S units contributed by the erythro-rich and S unit rich in β-O-4 structure [66]. Some kinds of pyrolysis products can be found in pyrogram (Figure 7)-derived S unit, G unit, and H unit with different relative abundance between lignin samples. The higher retention time indicated that the compound was degraded at a higher temperature. At a pyrolysis temperature of 350 °C, phenolic compounds such as eugenol (G6), aldehydes, ketones, or alcohol group from G- and S-unit were released. Other compounds such as vanillin (G7) and acetoguaiacone from G-unit were degraded at 200–400 °C, in which the β-ether was separated [67].

Table 3 shows pyrolysis products of reference, single-step, and fractionated-step lignin. The compounds detected by Py-GCMS are classified into five categories: aliphatic oxygen compounds and hydrocarbons, aromatic hydrocarbons, furan and phenol derivatives [68]. Based on pyrolysis products, single-step lignin had the highest total relative abundance of H unit followed by fractionated-step lignin and reference lignin. G unit presented the highest portion compared to the S and H unit in both isolated and reference lignin. The result indicated that the primarily pyrolysis composition of *A. mangium* kraft lignin from kraft black liquor of industrial pulp and paper was an S unit. This result is similar to wheat straw and pine sawdust lignin, mainly G unit. However, a different finding was reported in palm kernel shell (PKS) lignin, which was dominated by the H unit [69]. The classification of reference lignin was G-lignin, while single-step and fractionated-step lignin are classified as SGH-lignin. The total relative abundance of H and G units of single-step lignin was higher than that of fractionated-step. Inversely, the total relative abundance of the S unit of the single-step lignin was lower than fractionated-step lignin. H and G units are easier formed in terms of the possibility of forming condensation in the acid precipitation process. Based on this classification, the biphenyl bond of single-step lignin is relatively higher than fractionated-step lignin. The H unit does not have a methoxy group, and the G unit only has one methoxy group, leading to the formation of C-C biphenyl linkages. Thus, there is a higher yield of single-step lignin compared to fractionated-step lignin. The biphenyl linkages are included as covalent linkages, which are relatively more stable than β-O-4 ether linkages [68], so single-step lignin is relatively more stable and resistant to thermal treatment and biodegradation. The fractionated-step lignin had a total relative abundance of S unit higher than the single-step lignin, which showed that the fractionated-step lignin had more β-O-4 ether linkages and was more reactive than the single-step lignin.

### 3.7. Molecular Weights

**Figure 8:**
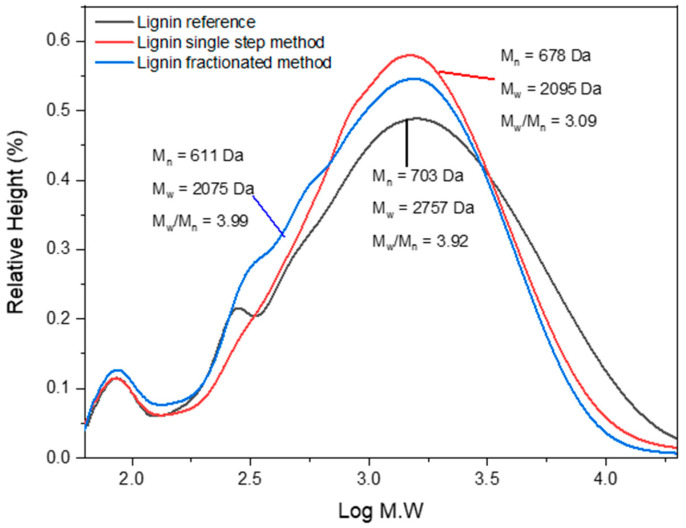
GPC curve including number-average (Mn), weight-average (Mw), and polydispersity index (PDI, Mw/Mn) of reference lignin, single-step lignin, and fractionated-step lignin.

The reactivity and physicochemical property are indicated by critical parameters such as molecular mass. The GPC curve in Figure 7 depicts the molecular weight of number-average (Mn), weight-average (Mw), and polydispersity index (PDI, Mw/Mn) of reference and isolated lignin from BL. The obtained molar mass distribution of lignin in this study is in the range of the Kraft lignin in a THF-based system reported by Baumberger et al. [70] (Mn = 200–2000 Da, Mw = 1500–50,000 Da). The number of Mw, Mn, and PDI depends on the isolation method, biomass source, and purification [71]. Figure 8 also shows that the trend of both Mw and Mn is reference lignin > single-step lignin > fractionated-step lignin. Markedly, the trend was similar to AIL content and lignin solubility in an organic solvent. However, the opposite trend is seen against the S/G ratio and total phOH. Reference lignin has a higher molecular mass, AIL content, and solubility in an organic solvent, yet it has a lower S/G ratio, Tg value, and total phOH than isolated lignin. This result contrasts with Gordobil et al. (2018), where a positive correlation between molecular weight, S/G ratio, and total phOH was observed. Different analytical methods may affect the S/G ratio and total phOH, ^31^P NMR vs Py-GCMS (S/G ratio) and UV-Vis (total phOH). Higher AIL content resulted in high molecular weight and ASL content due to different governing mechanisms of cleavage bonds and functional groups in lignin. This finding was similar to the results reported by Stiefel et al. [72], where the insoluble acid is slightly correlated (r^2^ = 0.739) with the molecular weight in lignin from different treatments [72].

The higher molecular weight of reference lignins was attributed to a higher percentage of the G unit [73]. This finding substantiated that reference lignins are derived from softwood. Higher PDI of fractionated-step lignin indicated wider molecular weight distribution as well as the existence of the impurities that positively correlated to ash content (Table 1) [52].

### 3.8. Future Potential of A. mangium Lignin from BL in Adhesive Applications for Wood-Based Composites

The structural features of *A. mangium* lignin extracted from BL exhibited a strong link to many alternative ways in its possible applications, according to the findings of this study. The fingerprint result (UATR-FTIR, ^1^H, and ^13^C NMR) and elucidation structure by Py-GCMS showed a higher abundance of G-unit which is the most active unit in phenolic resin polymerization. Besides, the high Tg value of isolated lignin is suitable for wood adhesive applications. The result was similar to lignin from coconut husk that was examined by Abd Latif et al. [74] as an alternative material for lignin-phenol-glyoxal adhesives. Markedly, high MW indicated higher content of aromatic protons which has a better chance of polymerization. Hence, lignin from the single-step method would be more suitable for wood adhesive applications. Another consideration is the presence of large amounts of phenolic hydroxyls in the isolated lignin structure which means making them reactive to create linkage with aldehyde [18].

## 4. Conclusions

This study investigated the chemical and physical properties of lignin derived from pulp mill factory residue (*Acacia mangium*) using diverse techniques. Lignin was successfully isolated through single-step and fractionated-step dilute acid precipitation. According to fingerprint analysis by FTIR, ^1^H, and ^13^C NMR, unique lignin peaks such as aromatic unit guaiacyl (G) and syringyl (S) were observed. The results were confirmed by the commercial lignin used as a reference. Dilute hydrochloric acid obtained high acid-soluble lignin (ASL) content. In contrast, fractionated-step lignin had lower lignin content than single-step. Still, it had a linear correlation against total phenolic hydroxyl (phOH) content, thermal stability, G-unit, and molecular weight distribution. More condensed G-unit in single-step lignin induced higher molecular weight distribution (Mw and Mn) and Tg value and total phOH. Single-step precipitation obtained the highest lignin yield, ~35.39 %. Comprehensive analysis of technical lignin aided in gathering knowledge of the structure and properties of lignin in suggesting better valorization strategies and enhanced future potential for wider industrial application of lignin as a renewable raw material.

## Figures and Tables

**Table 1:** Chemical composition of lignin.

	Water Content (%)	Ash Content (%)	AIL (%)	ASL (%)	Total phOH (mmol/g)
Lignin reference	2.60 ± 0.27	2.44 ± 0.00	96.02 ± 0.50	1.54 ± 0.06	6.00 ± 0.50
Lignin single method	5.65 ± 1.14	0.53 ± 0.07	77.45 ± 0.48	22.02 ± 0.83	7.40 ± 0.71
Lignin fraction method	15.79 ± 0.74	1.94 ± 0.08	69.94 ± 5.55	28.12 ± 0.94	7.31 ± 0.78

**Table 2:** Interpretation bands of UATR-FTIR spectra.

Code	Wavelength (cm^−1^)	Functional Group
a	3359	Hydroxyl group stretching (O-H) from aliphatic and aromatic [36]
b	2918	C-H stretching in methylene [33]
c	2854	C-H stretching in methoxy [33]
d	1710	Carbonyl (C=O) stretching in unconjugated aldehyde and ketone [34]
e	1590	C=C (aromatic rings) [34]
f	1511	C=C (aromatic rings) [34]
g	1470	Aromatic ring vibration with C-O [34]
h	1430	Deformation C-H in methyl group [4]
i	1326	C-O breathing (syringyl) [37]
j	1266	C-O(H) (phenolic OH guaiacyl) [36]
k	1213	C-O(Ar) in guaicyl ring [35]
l	1111	Deformation Ar-CH in syringyl ring [35]
m	1030	Unconjugated C-O in guaicyl [35]
n	855	CH out of plane bending in guaicyl [35]

**Table 3:** The list of pyrolysis product reference lignin, single-step lignin, and fractionated-step lignin.

Unit	Pyrolysis Product	Relative Abundance (%)			Fragmentation (*m/z*)
		Reference Lignin	Single-Step Lignin	Fractionated-Step Lignin	
**H1**	Phenol	1.02	2.76	3.58	94, 66, 45
**H2**	Phenol, 2-methyl-	1.18	1.39	1.32	108, 90, 79
**H3**	Phenol, 3 + 4-methyll	2.24	3.25	2.26	107, 90, 79
**H4**	Phenol, 2,4-dimethyl-	1.00	0.40	0.00	122, 107, 77
**H5**	Phenol, 4-vinyl	0.20	0.48	0.66	120, 91, 65, 40
**H6**	Catechol, 3-methyl	3.63	9.70	9.66	124, 78
**H7**	Catechol, 4-methyl	3.90	3.81	1.99	124, 78
**Total relative abundance of H unit**		**13.17**	**21.80**	**19.47**	
**G1**	Guaiacol	12.36	9.99	15.23	124, 109, 81
**G2**	Guaiacol-4-methyl-	18.62	13.49	5.96	138, 123, 95
**G3**	Guaiacol, 4-ethyl	7.02	5.19	3.13	152, 137
**G4**	Guaiacol, 4-vinyl	10.59	4.87	6.22	150, 135, 107, 77
**G5**	Guaiacol, 4-propyl	1.70	3.45	0.00	166, 137
**G6**	Eugenol	1.08	1.25	0.33	164, 149, 77
**G7**	Vanillin	5.57	1.36	1.80	151, 123, 109
**G8**	Isoeugenol (cis)	2.33	0.50	2.50	164, 149
**G9**	Isoeugenol (trans)	7.08	3.39	1.98	164, 149
**G10**	Acetoguaiacone	3.15	2.06	3.39	166, 151, 123
**G11**	Guaiacyl acetone	3.47	0.62	0.48	180, 137
**G12**	Propioguaiacone	1.70	0.00	1.23	180, 151, 123
**G13**	Dihydroconiferyl alcohol	7.30	0.00	0.00	182, 137
**G14**	Coniferyl alcohol	1.95	0.00	0.00	180, 137, 124, 91
**Total relative abundance of G unit**		**83.93**	**46.17**	**42.26**	
**S1**	Syringol	1.70	13.70	19.41	154, 139, 111, 96
**S2**	Syringol, 4-methyl	0.96	9.47	6.45	168, 153, 125
**S3**	Syringol, 4-vinyl	0.14	4.79	6.68	180, 165, 137
**S4**	Syringol, 4-propenyl (trans)	0.11	2.32	1.62	194, 179, 91
**S5**	Acetosyringone	0.00	2.24	4.12	196, 181, 153
**Total relative abundance of S unit**		**2.90**	**32.52**	**34.15**	
**S/G ratio**		**0.03**	**0.70**	**0.81**	

## Data Availability

The data presented in this study are available on request from the corresponding author.
